# Valinomycin Biosynthetic Gene Cluster in *Streptomyces*: Conservation, Ecology and Evolution

**DOI:** 10.1371/journal.pone.0007194

**Published:** 2009-09-29

**Authors:** Andrea M. Matter, Sara B. Hoot, Patrick D. Anderson, Susana S. Neves, Yi-Qiang Cheng

**Affiliations:** 1 Department of Biological Sciences, University of Wisconsin-Milwaukee, Milwaukee, Wisconsin, United States of America; 2 The Great Lakes WATER Institute, University of Wisconsin-Milwaukee, Milwaukee, Wisconsin, United States of America; 3 Department of Chemistry and Biochemistry, University of Wisconsin-Milwaukee, Milwaukee, Wisconsin, United States of America; Tata Institute of Fundamental Research, India

## Abstract

Many *Streptomyces* strains are known to produce valinomycin (VLM) antibiotic and the VLM biosynthetic gene cluster (*vlm*) has been characterized in two independent isolates. Here we report the phylogenetic relationships of these strains using both parsimony and likelihood methods, and discuss whether the *vlm* gene cluster shows evidence of horizontal transmission common in natural product biosynthetic genes. Eight *Streptomyces* strains from around the world were obtained and sequenced for three regions of the two large nonribosomal peptide synthetase genes (*vlm1* and *vlm2*) involved in VLM biosynthesis. The DNA sequences representing the *vlm* gene cluster are highly conserved among all eight environmental strains. The geographic distribution pattern of these strains and the strict congruence between the trees of the two *vlm* genes and the housekeeping genes, 16S rDNA and *trpB*, suggest vertical transmission of the *vlm* gene cluster in *Streptomyces* with no evidence of horizontal gene transfer. We also explored the relationship of the sequence of *vlm* genes to that of the cereulide biosynthetic genes (*ces*) found in *Bacillus cereus* and found them highly divergent from each other at DNA level (genetic distance values≥95.6%). It is possible that the *vlm* gene cluster and the *ces* gene cluster may share a relatively distant common ancestor but these two gene clusters have since evolved independently.

## Introduction

Microbial natural products mediate a wide range of biological and biochemical interactions both among microbes and between microbes and higher organisms [1], [2]. Information embedded in natural product biosynthetic genes and gene clusters includes not only biochemical codes for the production of diverse chemical molecules by enzyme catalysts, but also evolutionary and ecological clues about the origin, transmission and distribution of the genetic determinants of natural product biosynthesis. All genes are subject to evolution via vertical transmission (VT), through such processes as point mutation, gene duplication or deletion, recombination,or swapping. In addition to VT, natural product biosynthetic genes and gene clusters are often found to transmit intra- or inter-generically through lateral or horizontal gene transfer (HGT) as well [2], [3]. HGT origins are typically identified by criteria including incongruence between phylogenetic trees constructed with genes involved in primary metabolism [i.e. housekeeping genes such as 16S rDNA, *trpB* (encoding tryptophan synthetase β-subunit), *rpoC1* (encoding RNA polymerase β-chain)] and the genes in question, atypical sequence composition, the presence or absence of genes in closely related genera, and the flanking of genes by insertion elements such as transposons [4].

A classic example of HGT is the transmission of nonribosomal peptide synthetase (NRPS) genes responsible for the production of β-lactam antibiotics (e.g., penicillins and cephalosporins), evidently from bacteria to bacteria and from bacteria to fungi [5], [6]. Two recent surveys of polyketide synthase genes revealed the independent evolution of bacterial speciation and natural product biosynthetic genes, supporting the theory of HGT [7], [8]. In contrast, a study of microcystin biosynthetic genes by Rantala et al. [9] concluded that the microcystin biosynthetic genes from distantly related cyanobacteria appear to share an ancient common ancestor, and have co-evolved along with housekeeping genes for the entire history of toxin production. Furthermore, analysis of the conserved cyanopeptolin biosynthetic genes from three genera of cyanobacteria by Rounge at al. [10] identified independent evolution traces of those genes within each genus, disfavoring an origin by HGT.

Valinomycin (VLM; Figure S1) is a cyclic depsipeptide natural product with a wide range of reported biological activities including insecticidal, nematocidal, antibacterial, antiviral and apoptosis-inducing/cytotoxic/anticancer activities ([11] and references cited in [12]). Eleven *Streptomyces* strains isolated from around the world were reported to produce variable levels of VLM. The VLM biosynthetic gene cluster (*vlm*), cloned independently from *S. tsusimaensis* ATCC 15141 by us [12] and from *S. levoris* A9 by others [13], consists of two large NRPS genes (*vlm1* and *vlm2*, 10,286 bp and 7,967 bp, respectively) and a few functionally less defined small ORFs. Fifteen distinctive domains in the deduced NRPS mega-enzymes (Vlm1 and Vlm2) are organized into four modules (Figure S1), responsible for the incorporation of four substrate monomers: D-hydroxy-isovaleric acid, D-valine, L-lactic acid, and L-valine, respectively [13]. A C-terminal thioesterase domain on Vlm2 is postulated to mediate the oligomerization of three 4-unit intermediates to a linear full-length precursor and subsequently the cleavage and cyclization of precursor into the final product VLM [12].

Cereulide (Figure S1), an emetic toxin produced by a large number of *Bacillus cereus* strains [14], [15], is a natural analog of VLM. The cereulide biosynthetic gene cluster (*ces*), which is located on a mega virulence plasmid related to the *B. anthracis* toxin plasmid pXO1 [16], [17], shares a high degree of organizational similarity to that of the *vlm* gene cluster, and contains two large NRPS genes, *cesA* and *cesB*, which are highly homologous to *vlm1* and *vlm2* [13] (Figure S1). These observations prompt one to hypothesize an evolutionary relationship between the *ces* gene cluster and the *vlm* gene cluster.

Here we report a survey of the *vlm* gene cluster among diverse *Streptomyces* isolates and a phylogenetic comparison to the *ces* gene cluster. The objectives of this study were: 1) To determine whether all 10 strains of *Streptomyces* isolates in our sampling, including eight strains reported to produce VLM, and two non-producer strains, actually produce VLM; 2) To determine the placement of VLM-producing *Streptomyces* strains within an overall phylogenetic analysis of 16S rDNA data for 47 *Streptomyces* strains and two outgroups (*Mycobacterium tuberculosis* and *Nocardia farcinica*); 3) To explore whether the *vlm* gene cluster of diverse *Streptomyces* isolates from around the world display any evidence of HGT (as is common in other natural product biosynthetic genes) by comparing phylogenies based on the *vlm* DNA sequences with those based on the housekeeping genes, 16S rDNA and *trpB*; and 4) To determine how similar a representative *ces* gene cluster in *B. cereus* AH187 type strain is to the *vlm* gene cluster in *Streptomyces* at DNA level.

## Results

### Verification and Quantification of VLM Production

All 10 *Streptomyces* strains (Table 1) were subjected to unified fermentation conditions and their levels of VLM production were determined by liquid chromatography-mass spectrometry (LC-MS) (Figure S2). Positive ion signals were detected for multiple VLM adducts: [VLM+H]^+^ = 1111.6 *m/z*, [VLM+NH_4_]^+^ = 1128.7 *m/z*, [VLM+Na]^+^ = 1133.6 *m/z*, and [VLM+K]^+^ = 1149.8 *m/z*. Together, these signals form a unique, consistent, four-peaked fingerprint corresponding to VLM, which was detected in each of the eight *Streptomyces* strains previously reported to produce VLM, eluting at about 14 min. Peak areas were used to estimate the concentrations of VLM detected in each sample, in comparison to a commercially available standard (Sigma Aldrich, St. Louis, MO). VLM production ranges from 4.25 (*S. fulvissimus*) to 32.8 mg L^−1^ (*S. exfoliatus*). The VLM fingerprint was not detected in the negative control strain *S. coelicolor* or *S. hawaiiensis*.

**Table 1:** Bacterial strains used in this study, including acronyms, origin and whether to produce VLM.

Acronym	Full Name	Origin	VLM^c^	Source/Reference
S. TSUSI (JAP)	*Streptomyces tsusimaensis* (ATCC 15141)^a^	Japan	+	ATCC/[44]
S. spPRL (CAN)	*Streptomyces* sp. PRL 1642 (ATCC 23836)	Canada	+	ATCC/[45]
S. ANULA (USA)	*Streptomyces anulatus* (Montana)	USA	+	From the Pettit Lab/[46]
S. ANULA (MAL)	*Streptomyces anulatus* (Malaysia)	Malaysia	+	From the Pettit Lab/[46]
S. EXFOL (MAL)	*Streptomyces exfoliatus* (Malaysia)	Malaysia	+	From the Pettit Lab/[46]
S. FULVI (GER)	*Streptomyces fulvissimus* (DSM 40767)	Germany	+	DSMZ/[47]
S. GRIS1 (FIN)	*Streptomyces griseus* 1/k (DSM 41748)	Finland	+	DSMZ/[26]
S. GRIS2 (FIN)	*Streptomyces griseus* 10/ppi (DSM 41751)	Finland	+	DSMZ/[26]
S. COELI	*Streptomyces coelicolor* A3(2) (ATCC BAA-471)^b^	(N/C)	−	ATCC/[21]
S. HAWAI	*Streptomyces hawaiiensis* (NRRL 15010)^b^	(N/C)	−	NRRL/[48]

a Reference strain;

b Negative control strains;

c + or − indicates whether or not the strain produces VLM; N/C, not concerned.

### Sampling of VLM Biosynthetic Gene Fragments

**Figure 1:**
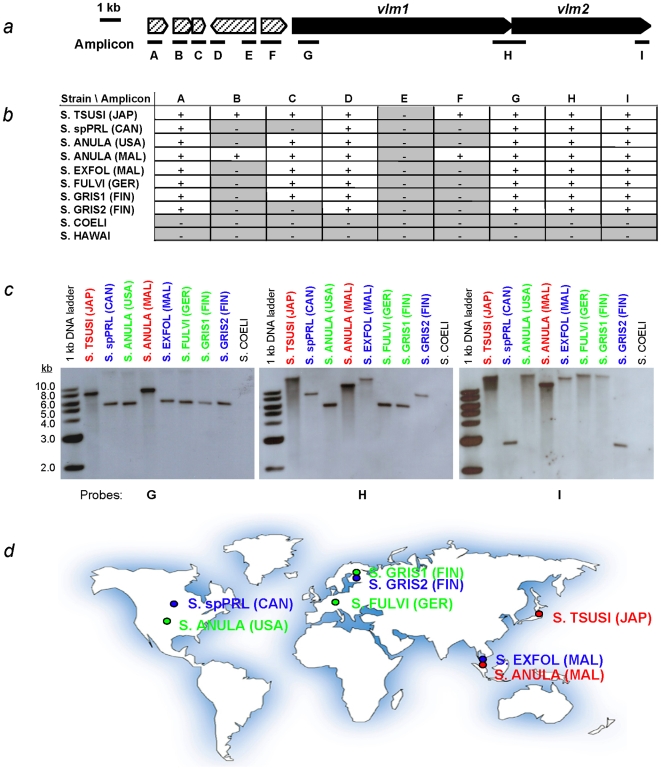
VLM biosynthetic gene cluster conservation and distribution. (*a*) The *vlm* gene cluster from *S. tsusimaensis* including two critical nonribosomal peptide synthetase (NRPS) genes (*vlm1* and *vlm2*) and five ORFs [12], and the position of nine regions targeted by DNA amplification (amplicons A to I; also see Figure S1). Amplicons G, H and I were used as molecular probes for downstream experiments. (*b*) Checkerboard of amplicons that were either amplified successfully by PCR (+, white background,) or not (−, grey background) from test strains (by acronyms. See Table 1 for full names and other information). (*c*) Southern hybridization patterns of *Streptomyces* genomic DNAs probed by Amplicon G, H or I, respectively. Strain S. COELI (by acronym) serves as a negative control. Strains identified in a clade during phylogenetic analyses (Fig. 3) are labeled in one color. Approximately 2 µg of total DNA from each strain was digested with *Pst*I. (*d*) Geographic distribution of the VLM-producing strains around the world with the same color-coded phylogenetic relatedness.

Of the nine regions (amplicons A to I) targeted for PCR amplification based on homology to the *vlm* gene cluster of reference strain *S. tsusimaensis* [12] (Fig. 1a), five regions were successfully amplified from all eight VLM-producing strains; none was amplified from either negative control strains (Fig. 1b). The five amplified regions, corresponding to the reference gene cluster, include: ORF11 (encoding a putative necrosis-inducing factor) (amplicon A), the 3′ region of ORF14 (function unknown) (amplicon D); the 5′ region of *vlm1* (1,113 bp, encoding an adenylation domain in Vlm1 NRPS module 1), designated “*vlm1*” (amplicon G; also see Figure S1); the region spanning the 3′ region of *vlm1* (766 bp, encoding part of a condensation domain in Vlm1 NRPS module 3), a short intergenic region (23 bp) and the 5′ region of *vlm2* (1,038, encoding part of an adenylation domain in Vlm2 NRPS module 1), designated “*vlm1/2*” (amplicon H; also see Figure S1), and the 3′ region of *vlm2* (668 bp, encoding a terminal thioesterase domain), designated “*vlm2*” (amplicon I; also see Figure S1). Since the functions of the putative ORFs are not yet established, only the DNA sequences of last three amplicons (G, H and I) were used for phylogenetic analyses and Southern hybridization. DNAs of near-complete 16S rDNA and *trpB* were successfully amplified from all strains used in this study.

### Detection of VLM Biosynthetic Gene Homology by Southern Hybridization

All eight VLM-producing strains (including reference strain *S. tsusimaensis*) tested positive for genes *vlm1* and *vlm2*, as well as the “*vlm1/2*” intergenic region; the control strain, *S. coelicolor*, tested negative (*S. hawaiiensis* not included due to lack of gel space) (Fig. 1c). Hybridized restriction fragment lengths evidently clustered among the *vlm*-positive strains. Probe G hybridized to two clear classes of fragment, 5.5 kb and 9.0 kb in size; Probe H, which spans the 3′ region of *vlm1* through the 5′ region of *vlm2*, hybridized to fragments with three size ranges: 6.0 kb, 9.0 kb and >10 kb; and Probe I hybridized to two or three classes of fragment ranging in size from 3.0 kb to >10 kb. Those hybridization patterns are color-labeled and have been found largely in agreement with the following phylogenetic data (see Phylogenetic Analyses), except for strain *S. exfoliatus* (Malaysia) which deserves more discussion later. Those results suggest that the *vlm* gene cluster is highly conserved among all VLM-producing strains isolated from a wide range of geographic locations, as probes hybridized during high stringency washes (2×15 min in 0.2×SSC, 0.1% SDS at 68°C), indicating significant sequence identity. Pairwise sequence comparison indicated that *vlm* amplicons of the same region from all eight VLM-producing strains have greater than 86% of actual sequence identity (Figure S3).

### Phylogenetic Analyses

#### *Streptomyces* 16S rDNAs

**Figure 2:**
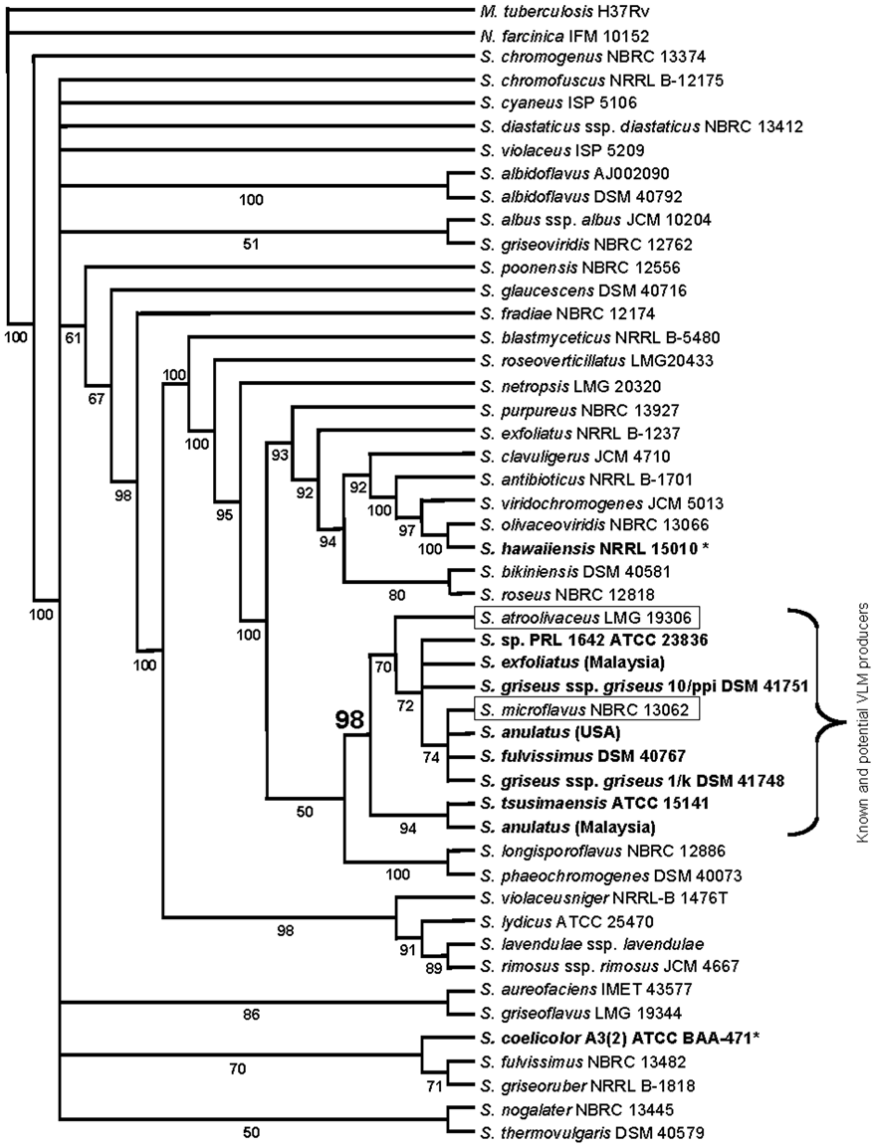
Bayesian inference (BI) majority rule consensus tree of *Streptomyces* strains. A sampling of 47 randomly selected *Streptomyces* strains and two outgroups are analyzed by 16S rDNA sequences. Each supported node is labeled with its posterior probability. Strains used in this study are in bold-face type, including negative controls (^*^).

The 16S rDNA sequences of a total of 49 taxa, including 10 strains obtained by this study, 37 randomly selected *Streptomyces* strains and two outgroups, were analyzed using MP and BI in an effort to determine how the VLM-producing strains are distributed throughout *Streptomyces* (Fig. 2; also see Table S3 for detailed parameters). The alignment length is 1413 bases with five indels that were treated as missing data and not scored. Individual sequences range in length from 1376 bp (*S. chromogenus* NBRC 13374) to 1393 bp (*M. tuberculosis* H37Rv). MP analysis resulted in 99 equally parsimonious trees; the MP strict consensus and bootstrap trees (not presented) were largely congruent with the BI majority rule consensus tree, especially where bootstrap (BS) or posterior probability (PP) values were moderate to high. While the topology of the non-VLM-producing *Streptomyces* differs somewhat between the MP and BI generated trees, the clade containing the VLM-producers is identical and moderately supported as monophyletic (BS = 76, PP = 98).

Within this clade, *S. tsusimaensis* and *S. anulatus* (Malaysia) are sister to a largely unresolved clade consisting of all remaining VLM-producers and two additional *Streptomyces* strains (*S. atroolivaceus* LMG 19306 and *S. microflavus* NBRC 13062) not known to produce VLM.

#### VLM biosynthetic genes

**Figure 3:**
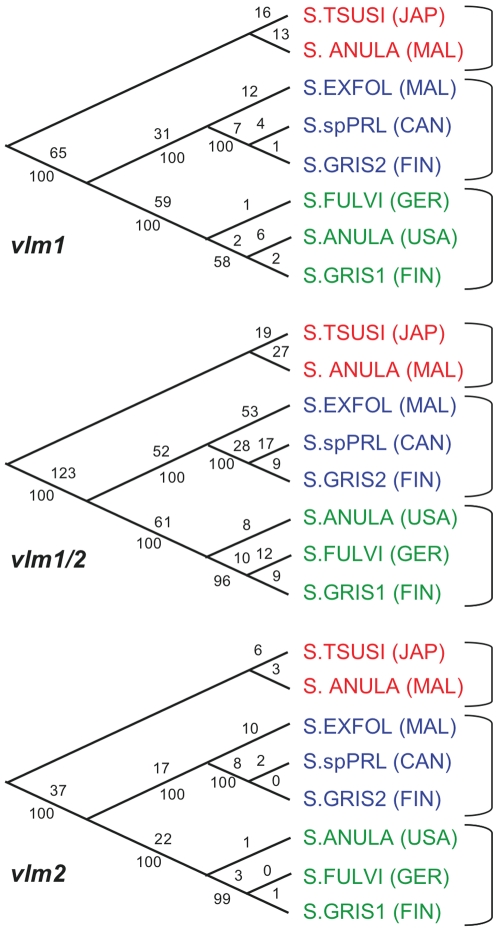
Maximum parsimony (MP) analysis of *vlm* sequences. Analysis of *vlm1*, *vlm1/2* and *vlm2* sequences from all eight VLM-producing strains generated almost identical phylogenetic trees, indicating the representability of short gene fragments for not-so-easily accessible sequences of large natural product biosynthetic genes (e.g. NRPS genes).

The data for *vlm1*, *vlm1/2* and *vlm2*, were analyzed separately, using MP. The trees produced had very similar topologies (Fig. 3 and Table S3). All trees were midpoint rooted since an appropriate outgroup is not available for *vlm* data. In all three trees, grouping of strains (BP = 100) are consistent: *S. tsusimaensis* and *S. anulatus* (Malaysia) formed a clade sister to the remaining *Streptomyces* species, *S. exfoliatus* are sister to *S.* sp. PRL1642 and *S. griseus* 10/ppi, and the rest group together with slightly variable topologies.

**Figure 4:**
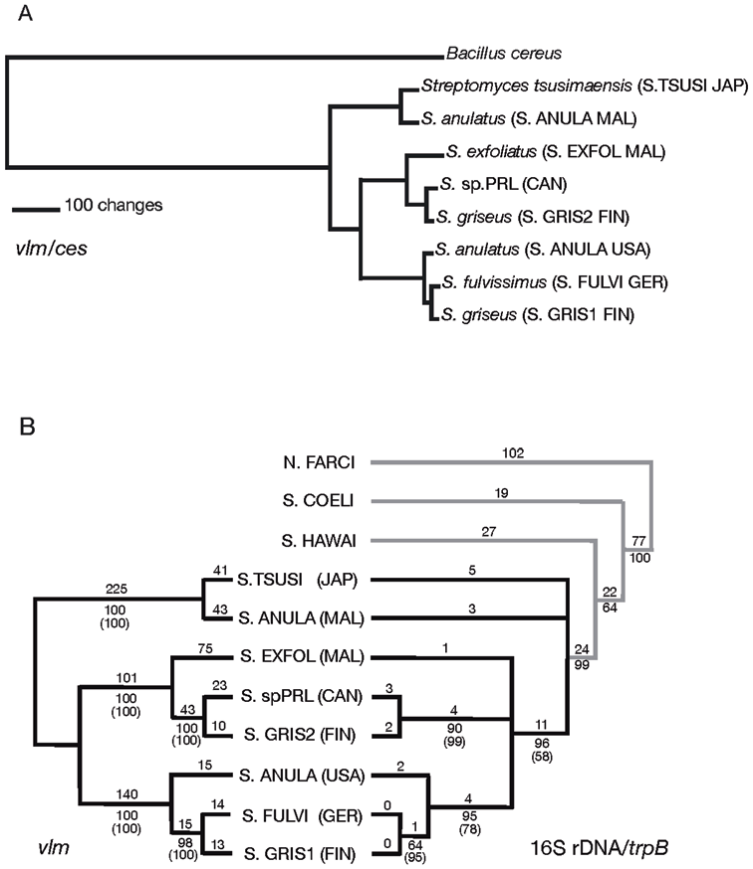
Phylogenetic analyses of *vlm* sequences with *ces* sequences and with housekeeping gene sequences. (A) ML phylogram resulting from analysis of *vlm/ces* data for *Streptomyces* strains and a *Bacillus cereus* type strain AH187. (B) Strict consensus trees resulting from maximum parsimony (MP) analyses of combined *vlm* (left half) data and combined 16S rDNA/*trpB* (right half) data. The *vlm* data set is mid-point rooted; the 16S rDNA/*trpB* data set is outgroup rooted with *Nocardia farcinica*. Branch lengths appear above the branches; bootstrap values (1000 replicates) and Bayesian inference (BI) analysis posterior probability values (parentheses) appear below. Species acronyms are as in Fig. 4A.

The partition homogeneity test for *vlm1*, *vlm1/2*, and *vlm2* supported the congruence and subsequent combination of the three gene segments (P = 0.259). Also, the trees from different regions of the *vlm* genes are similar in topology (Fig. 3); therefore the data sets were combined for MP and BI analyses. Both model test algorithms chose GTR+G [18], [19] as the best fitting model for individual *vlm* sequences; therefore, there was no need to partition data when running BI on the combined data set. The overall topology of both the MP and BI trees produced from the combined *vlm* data are congruent with the trees produced from individual data sets, recognizing the same three clades (Fig. 4B, left half). All support values of the combined *vlm* data were extremely high (BS≥98, PP = 100).

The translated protein sequences of the *vlm* data sets (Vlm1, Vlm1/2, Vlm2 and Vlm-combined) were also analyzed by MP in order to confirm the robustness of the nucleotide MP analysis and to identify a potential outgroup for rooting purposes. The topologies of the resultant trees are similar to those from the nucleotide data (trees not presented). Specifically, MP analysis of the combined Vlm protein data set produced two equally parsimonious trees with low homoplasy (see Table S3 for detailed parameters). When the tree was outgroup rooted, the topology of the combined Vlm protein data set was the same as for the nucleotide data, except for the third clade, which collapsed in the strict consensus tree to form a trichotomy. Bootstrap values for supported branches are all 100%, not including the *S. anulatus* (USA)/*S. griseus* 1/k branch, which is weakly supported (BS = 58).

#### Assessing similarity of the *vlm* (*Streptomyces*) and the *ces* (*Bacillus cereus*) gene clusters

The F81 pairwise analysis of genetic distances demonstrated that the *vlm* sequences of all *Streptomyces* strains are relatively distant from the representative *ces* sequence of *B. cereus* AH187 (Table S4). All *vlm* sequences sampled had genetic distances ranging from 0.82–12.8%; whereas the distances between *vlm* and *ces* sequences ranged between 95.6–98.9%, suggesting that the *vlm* gene cluster and the *ces* gene clusters evolved independently for a relatively long period of time. Aligning the *Bacillus ces* sequence with *Streptomyces vlm* sequences was actually very difficult and presented many homology issues. Because of this, we included the *ces* sequence of *B. cereus* in our ML analyses only to give some idea of the long branch that separates the *ces* data from all *Streptomyces vlm* sequences (Fig. 4A) and did not employ *ces* sequence as an outgroup for *vlm* analyses (Fig. 3 and Fig. 4B).

#### 16S rDNA and *trpB* of the VLM-producing strains

The 16S rDNA and *trpB* gene sequences of eight VLM-producing strains, along with those of negative control strains, were analyzed separately using MP to determine the phylogeny reflective of *Streptomyces*, to assess its congruence with that of the *vlm* gene fragments. All phylogenies are outgroup rooted with *Nocardia farcinica*. In the MP tree of 16S rDNA data set (tree not presented), the VLM-producing strains form a well supported clade (BS = 96), with *S. tsusimaensis* sister to all remaining strains. Within this grouping, *S. anulatus* (Malaysia) is sister to a basal polytomy consisting of *S.* sp. PRL 1642, *S. exfoliatus*, *S. griseus* 10/ppi and an unresolved clade consisting of the three remaining VLM-producing strains.

The MP analysis of the *trpB* data resulted in seven equally parsimonious trees (Table S3). In the MP strict consensus tree, all of the *Streptomyces* strains, non-VLM and VLM-producers group in a well supported (BS = 100), unresolved polytomy (tree not presented). Within this large polytomy the following group as sister species: *S. coelicolor* with *S. hawaiiensis* (non-VLM producers); *S. tsusimaensis* with *S. anulatus* (Malaysia); *S.* sp PRL 1642 with *S. griseus* 10/ppi; and *S. anulatus* (USA) sister to *S. fulvissimus* and *S. exfoliatus*.

Because both the 16S rDNA and *trpB* data sets produced unresolved MP trees, the data sets were then combined even though the P-score for the partition homogeneity test was low (P = 0.01; see Table S3) in order to increase resolution. A known weakness of the partition homogeneity test is incorrect rejection of the null hypothesis when the number of informative sites is low [20]. Furthermore where individual trees are resolved, especially with moderate to excellent support (BS≥70), the topology is similar between the 16S rDNA and the *trpB* trees. MP analysis of the combined data resulted in five equally parsimonious trees. In the MP strict consensus tree, all of the VLM-producers form a monophyletic group with *S. tsusimaensis* and *S. anulatus* (Malaysia) forming a basal trichotomy with a clade consisting of all remaining VLM-producers. *S. exfoliatus*, the sister species *S.* sp. PRL 1642 and *S. griseus* 10/ppi, and a clade consisting of *S. anulatus* (Malaysia), *S. fulvissimus* and *S. griseus* 1/k, form a trichotomy as well (Fig. 4B, right half).

BI analysis was also performed on the 16S rDNA/*trpB* combined data set to compare with the results of MP analysis. The best fitting nucleotide substitution model chosen by both hLRTs and AIC for the 16S rDNA data set is GTR+I+G, while the best fitting model for *trpB* is GTR+I [18], [19]. Therefore, the combined data set was partitioned to reflect these differing models. The majority rule consensus tree has a similar topology to that of the MP tree (tree not presented), but *S. tsusimaensis* and *S. anulatus* (Malaysia) are included in a weakly supported clade (PP = 0.56) with *S. coelicolor* and *S. hawaiiensis*.

#### Combined *vlm*/16S rDNA/*trpB* of the VLM-producing strains

All three *vlm* data sets were combined with 16S rDNA and *trpB* data for MP and BI analysis, supported by a strong P-score of 0.405 for the partition homogeneity test (Table S3). This was done to substantiate congruence between the *vlm* data with that of the housekeeping genes, 16S rDNA and *trpB*. MP and BI analyses produced unrooted trees (not presented) with the same topology as the combined *vlm* data (Fig. 4B) and with highly supported branches (BS≥99 and PP = 1.00).

### Other Analyses

The *vlm1* and *vlm2* datasets have a slightly lower G+C content (69 and 67%, respectively) than that of *vlm1/2* (71%), but the G+C content (70%) of the combined *vlm* sequence is in close proximity to what is typical for *Streptomyces* (e.g. 72% in *S. coelicolor* [21]) (Table S5). Furthermore, the *vlm* data sets were analyzed for non-synonymous versus synonymous substitution rates using the SNAP program [22]. Calculations of ds (synonymous) to dn (non-synonymous) substitutions included: 0.60 (*vlm1*), 1.72 (*vlm1/2*), 1.34 (*vlm2*) and 1.57 (*vlm*-combined).

Pulse field gel electrophoresis and subsequent Southern hybridization with the amplicon G probe indicates that the *vlm* gene cluster is on the chromosome of every VLM-producer and not on a plasmid (data not shown). This is in agreement with the findings by [23] who likewise reported that the putative *vlm* gene cluster of *S. levoris* A9 is present on the chromosome and not on a plasmid.

## Discussion

The *vlm* gene cluster (Fig. 1a, also see Figure S1), represented by the three DNA regions surveyed in this study, is highly conserved among all eight VLM-producing strains. Strong similarity is indicated by the ability to amplify all three regions of the *vlm* genes from all taxa (except negative control strains) by PCR using primers based on the sequences of reference strain *S. tsusimaensis* (Fig. 1b). Strong similarity among the *vlm* genes of VLM-producing strains is also supported by the positive results of Southern analysis. Each of the three regions was detected in all VLM-producing strains under conditions of high stringency (Fig. 1c). Restriction fragment length patterns also generally correlate with the three clades: 1) *S. tsusimaensis* and *S. anulatus* (Malaysia), 2) *S.* sp. PRL1642 and *S. griseus* 10/ppi, and 3) *S. anulatus* (USA), *S. fulvissimus* and *S. griseus* 1/k. The remaining one, *S. exfoliatus* (Malaysia), groups variably with others; its pattern is similar to the clades 2) and 3) when probed for *vlm1* (amplicon G), close to the clade 1) when probed for *vlm1/2* (amplicon H), but more similar to the clades 1) and 3) when probed for *vlm2* (amplicon I). Variations of the restriction fragment length patterns may attribute to very recent point mutations that have altered certain restriction enzyme sites.

VLM was detected by LC-MS from all strains verified to contain the *vlm* gene cluster, thus indicating that the gene cluster is intact and was actively expressed in every strain under the conditions tested. Fermentation conditions were not optimized for individual strains; therefore, the levels of VLM production were relative. The active production of this antibiotic by all strains containing the gene cluster implies that VLM must confer certain selective advantages in nature. Nevertheless, the *vlm* gene cluster is not essential. Our previous studies have generated *vlm–*gene insertion mutants which completely lost VLM production but had no apparent defect in growth or differentiation [12]. VLM was repeatedly discovered for its agricultural and potential pharmaceutical applications ([11] and references cited in [12]). It remains intriguing yet difficult to probe the indigenous function of VLM in *Streptomyces*.

Despite a significant structural similarity between VLM and cereulide and an organizational similarity between the *vlm* gene cluster and the *ces* gene cluster [13] (Figure S1), our analyses did not identify a close relationship between these two gene clusters. The fact that the overall G+C content of the *vlm* gene cluster of *Streptomyces* is about 70% [12] and that of the *ces* gene cluster of *B. cereus* is about 36% [16], [17], each of which falling within the typical G+C content range of the organism that harbors the gene cluster, suggests that the codon usage in these two groups of bacteria is very different. It is possible that the *vlm* gene cluster and the *ces* gene cluster may share a relatively distant common ancestor but these two gene clusters have since evolved independently.

Combined analysis of the nucleotide sequence from the three *vlm* gene regions, *vlm1*, *vlm1/2* and *vlm2*, resulted in a more robust phylogeny than individual data sets using MP and BI analyses. The differing relationships of *S. griseus* species, *S. fulvissimus*, and *S. anulatus* (USA) among the various gene regions are weakly supported, probably due to a low number of evolutionary changes between the taxa. Within the Vlm1 protein data set in particular, zero to two character states define the *S. anulatus* (USA)/*S. fulvissimus* relationship. The amount of parsimony informative characters is substantially increased during combined analysis, thus improving the overall accuracy of the phylogenetic inference.

MP and BI analyses of the individual and combined 16S rDNA and *trpB* data sets resulted in less resolved phylogenies, although the consensus trees are congruent with the combined *vlm* sequence MP and BI trees where resolved (Fig. 4B). 16S rDNA has been known to be too conserved to resolve relationships among closely related strains [24]. Concatenating 16S rDNA sequence with other housekeeping genes such as *trpB* had previously resolved relationships between *Streptomyces* species with success [25]. Nevertheless, the additional character information from *trpB* is not enough to completely resolve the relationships between the highly related strains in this study, but it does provide independent support about the correct root of the *Streptomyces* tree and additional support for clades.

Surprisingly, the VLM-producers do not group by geographic origin (Fig. 1d). Most notably, *S. griseus* 1/k and 10/ppi, which were isolated from the same area [26], occur in separate clades. Based on MP analysis of the combined *vlm* sequences, these two strains differ by at least 322 characters, as opposed to less than 100 characters between them and their nearest neighbors (both different species). A similar situation is seen between the *S. anulatus* strains (Malaysia and USA). As microbial distribution is a result of both environmental and dispersal processes [27], it remains unclear how these taxonomically closely related bacterial strains dispersed around the globe while two isolates from the same area are phylogenetically distinct from each other based on both housekeeping genes and secondary metabolic genes. These observations warrant revisiting the taxonomic definition of some of the VLM-producers, as many of the strains were defined decades ago.

Evolutionary patterns within natural product biosynthetic genes and gene clusters generally show strong evidence of HGT, supported by certain signatures such as incongruence between phylogenetic trees of housekeeping genes versus the gene in question [4]. Although the resolution of the phylogenetic tree produced from the 16S rDNA/*trpB* combined data set is not as robust as that produced from the combined *vlm* data set, the tree reveals complete congruence with the phylogenetic tree of the *vlm* sequences (Fig. 4B). Furthermore, the partition homogeneity test between the *vlm* data sets and the housekeeping genes resulted in a P-value of 0.405, indicating statistical congruence [28]. Both results suggest that the *vlm* genes represent organismal evolution by VT not HGT. In other words, the *vlm* genes have co-evolved along with the housekeeping genes, at least in a more recent evolutionary timeframe.

A similar, but less resolved topology with the VLM-producers was found when sampling the 16S rDNA of many more *Streptomyces* taxa (Fig. 2). This larger 16S rDNA tree was constructed to represent the distribution of *Streptomyces* species throughout the genus. The tree reveals that the VLM-producing *Streptomyces* are not randomly distributed, but rather found in a well-supported monophyletic clade (BS = 76, PP = 98) that includes two additional *Streptomyces* strains. Because of their presence in this grouping, *S. atroolivaceus* LMG 19306 and *S. microflavus* NBRC 13062, were recently obtained from public stock centers but tested negative for both VLM production and the presence of *vlm* gene fragments (data not shown). It is not clear whether these two strains have never had the gene cluster, or if they have completely lost the *vlm* gene cluster. The monophyletic grouping of the *vlm* gene cluster within the *Streptomyces* species is highly suggestive of inheritance from a common ancestry by VT rather than by HGT, which would typically result in a more sporadic distribution throughout the trees, amongst less highly related strains [29].

## Materials and Methods

### Sampling, Bacterial Strains and Cultivation Conditions

Our sampling includes eight available VLM-producing *Streptomyces* strains (out of 11 strains reported in the literature) isolated from diverse geographic locations around the world, and two non-producer control strains (Table 1; Fig. 1d). These *Streptomyces* strains were obtained either from public culture collections or from the Pettit laboratory. Strains were grown from spore suspensions (stored at −80°C) in tryptic soy broth (TSB) supplemented with 34% (w/v) glucose and 0.5% (w/v) glycine in shake flasks containing glass beads to break apart mycelia. Cultures were incubated at 30°C with agitation at 150 rpm for approximately 60 hr before harvested for total DNA preparation by Kirby mix procedure described in [30].

### Valinomycin Detection

VLM production was detected and quantified by LC-MS, similarly to previously described [12]. All 10 *Streptomyces* strains were grown in 50 ml of fermentation medium at 30°C for 6 days under constant agitation (150 rpm). Cells and resins (Diaion HP-20 from Supelco, Bellefonte, PA) were then collected together by centrifugation at 4,000×g for 20 min at ambient temperature and lyophilized to dryness. Crude VLM preparation was obtained by extracting the dried cell debris and resins with 25 ml methanol. Twenty µl of this preparation was injected into an 1100 Series LC/MSD Trap mass spectrometer (Agilent, Santa Clara, CA) for detection of the positive ion signals of VLM. The LC program included a 5-min linear gradient from buffer A (50% acetonitrile with 0.1% formic acid) to buffer B (99.9% acetonitrile with 0.1% formic acid), a constant elution in buffer B for 10 min, followed by a linear gradient return to buffer A in 5 min. Samples were fractionated by a 2.1×50 mm Eclipse Plus-C18 column (Agilent) with a flow rate of 0.5 ml min^−1^.

### Primer Design and Sequence Analyses

Primers (see Table S1 for details) were designed to amplify and sequence *vlm* gene fragments based on conserved regions of protein sequences. Protein homologies were identified by protein-protein BLAST (blastp), position-specific iterated and pattern-hit initiated BLAST (PSI- and PHI-BLAST) using the published *vlm* gene cluster sequence (GenBank accession no. DQ174261) of *S. tsusimaensis* as reference. Subsequent protein sequences were aligned using ClustalW [31] as part of the LaserGene software package (DNAStar, Madison, WI). Sequences for individual isolates were aligned using SeqMan. Individual chromatograms were inspected for integrity and ambiguous bases were corrected when there was a sequence overlap. Consensus sequences for individual isolates were exported, edited with SeqEdit (when concatenated to another data set), and imported into MegAlign for alignment under the criteria set by ClustalW. Alignments were manually inspected before exporting in the Nexus format [32]. Additional corrections were made using McClade 4, v. 4.01 [33], including end trimming to remove areas of missing data, and further alignment of indels (insertions and deletions) before phylogenetic analysis.

### PCR, DNA Sequencing and Southern Analysis

A typical PCR reaction contained 50–100 ng of total DNA, 7.5% DMSO, 100 µM of each dNTP, 1×ThermoPol buffer, 2.5 units of *Taq* DNA polymerase (New England BioLabs, Ipswich, MA), and 0.5 µM of each primer (Operon Biotechnologies, Huntsville, AL) in a total volume of 50 µl. PCR was carried out at multiple program conditions with multiple sets of primers in order to optimize amplification for each gene fragment. Weakly amplified DNAs were cloned into pGEM–T Easy vector (Promega, Madison, WI) for propagation. DNA sequencing was performed at the University of Wisconsin-Madison Biotechnology Center. Three probes were prepared from purified PCR products of *S. tsusimaensis* that include amplicons G, H and I (Fig. 1a). Labeling of DNA as probe, Southern blotting, hybridization and detection were performed per manufacturer's instructions, using the DIG High Priming DNA Labeling and Detection Starter Kit II (Roche Diagnostics, Indianapolis, IN).

### Phylogenetic Analyses

The 16S rDNA sequences of the eight VLM-producing strains obtained by this study, together with those of two negative control strains [*S. hawaiiensis* NRRL 15010 and *S. coelicolor* A3(2)], and 37 randomly selected *Streptomyces* strains and two outgroups (of *Mycobacterium tuberculosis* H37Rv and *Nocardia farcinica* IFM 10152) that were obtained from The Ribosomal Database Project (RDP-II) [34] or the GenBank database (see Table S2 for GenBank accession numbers), were analyzed using MP and BI in an effort to determine how the VLM-producing strains are distributed throughout *Streptomyces*.

Data sets were analyzed independently and in combination for phylogenetic reconstruction using the analytical methods maximum parsimony (MP) and Bayesian inference (BI). MP analyses were performed using PAUP* 4.0b10 [35], employing the branch and bound search option for all data sets except for those containing greater than 20 taxa; for these a heuristic search using 100 random addition sequence replicates was employed. Since indels were not plentiful and the additional information did not improve tree resolution, gaps were treated as missing data and not scored. Outgroups were chosen based on homology and ease of sequence alignment in order to root trees. In cases where outgroups were unavailable (for *vlm* gene nucleotide data), trees were midpoint rooted. If multiple best trees were produced, a strict consensus tree was calculated. Bootstrapping calculations were performed with 1000 replicates and a branch and bound search for the *Streptomyces* 16S rDNA data set, for which a heuristic search with 100 replicates was used due to the large size of the data set (40+ taxa, 1300 bp each). Consistency indices (measuring degree of homoplasy) were calculated excluding uninformative characters to prevent artificially inflated values [36].

Before combining any data sets, a partition homogeneity test (otherwise known as the incongruence length difference test) [37], was implemented in PAUP* 4.0b10 using 1000 branch and bound search replications to determine compatibility. This test measures the character congruence of two or more data sets. The null hypothesis of congruence is accepted (P≥0.05) by calculating the probability that the sum of the most-parsimonious tree lengths derived from random partitions of the data sets is equal to or lower than the sum of the tree lengths from the individual data sets. If the probability is low (P≤0.05), i.e. the random partitions have more homoplasy than the individual data sets, then the null hypothesis of congruence is rejected. When the null hypothesis is rejected, the data sets are considered incongruent and are not necessarily suitable for combination.

BI analyses were conducted using MrBayes 3.1 [38]. Best-fit substitution models for individual data sets were identified using the hierarchical likelihood ratio test (hLRT) and the Akaike Information Criterion (AIC) implemented in MrModelTest v2.2 [39]. Data were partitioned if different models were identified for individual data sets before combination. Parameters were set for the general model of DNA substitution [general time reversible (GTR); nst = 6] with different rate variations (as determine by hLRT and AIC), and otherwise flat priors. Markov chains (four, three heated) were run for one or two million generations, sampling trees every 100 generations for a total of 10,001 or 20,001 samples. Stationarity was determined to have been reached at 10% of the total sample size; therefore the first 1,000 or 2,000 trees were discarded as the burn-in phase. The standard deviation of split frequencies and parameter values were checked [potential scale reduction factor (PSRF) = 1.0] to verify that the analyses had been run long enough. Majority rule consensus trees were summarized and exported, showing posterior probabilities and average branch lengths.

To assess the similarity of a representative *ces* gene cluster in *B. cereus* AH187 type strain to the *vlm* gene cluster in *Streptomyces*, concatenated sequence regions of *vlm* genes were compared to those of *ces* genes. Alignments were accomplished using ClustalW [31], with subsequent manual alignment by eye in McClade 4, v. 4.01 [33]. Genetic distances were calculated using PAUP* and the F81 algorithm [40], which accounts for the unequal base frequencies of our data [41]. To show branch lengths via a phylogram, an ML analysis was performed with Garli vers. 0.95 [42], using the default parameters with “save every improved topology” unchecked.

### Other Analyses

G+C content and sequence divergence calculations were performed using shareware programs respectively: GCUA (General Codon Usage Analysis) [43] and SNAP (Synonymous Non-synonymous Analysis Program; www.hiv.lanl.gov) [22]. Pulse field gel electrophoresis was performed on all VLM-producing strains according to the manufacturer's instructions (Bio-Rad, Hercules, CA).

## Supporting Information

Table S1 Primers used to amplify the VLM biosynthetic gene (vlm) fragments, 16S rDNA and trpB.
(0.08 MB DOC)

Table S2 Strains and GenBank accession numbers of DNA sequences used for phylogenetic analyses.
(0.14 MB DOC)

Table S3 Primary data of phylogenetic analyses.
(0.09 MB DOC)

Table S4 Genetic distances calculated between concatenated vlm (Streptomyces) and ces (Bacillus cereus) DNA sequences.
(0.05 MB DOC)

Table S5 G+C content and substitution rates for the vlm data sets.
(0.04 MB DOC)

Figure S1 Structures of valinomycin and cereulide, and the domain/module organization and substrate specificity of their respective NRPSs.
(0.40 MB TIF)

Figure S2 Verification and quantification of VLM production.
(0.13 MB TIF)

Figure S3 Percentage identity and divergence of pairwise comparison among the vlm sequences.
(1.32 MB TIF)

## References

[1] Dixon N, Wong LS, Geerlings TH, Micklefield J (2007). Cellular targets of natural products.. Nat Prod Rep.

[2] Fischbach MA, Walsh CT, Clardy J (2008). The evolution of gene collectives: How natural selection drives chemical innovation.. Proc Natl Acad Sci U S A.

[3] Lawrence J (1999). Selfish operons: the evolutionary impact of gene clustering in prokaryotes and eukaryotes.. Curr Opin Genet Dev.

[4] Koonin EV, Makarova KS, Aravind L (2001). Horizontal gene transfer in prokaryotes: quantification and classification.. Annu Rev Microbiol.

[5] Liras P, Rodriguez-Garcia A, Martin JF (1998). Evolution of the clusters of genes for beta-lactam antibiotics: a model for evolutive combinatorial assembly of new beta-lactams.. Int Microbiol.

[6] Brakhage AA, Al-Abdallah Q, Tuncher A, Sprote P (2005). Evolution of beta-lactam biosynthesis genes and recruitment of trans-acting factors.. Phytochemistry.

[7] Ridley CP, Lee HY, Khosla C (2008). Evolution of polyketide synthases in bacteria.. Proc Natl Acad Sci U S A.

[8] Metsa-Ketela M, Halo L, Munukka E, Hakala J, Mantsala P (2002). Molecular evolution of aromatic polyketides and comparative sequence analysis of polyketide ketosynthase and 16S ribosomal DNA genes from various streptomyces species.. Appl Environ Microbiol.

[9] Rantala A, Fewer DP, Hisbergues M, Rouhiainen L, Vaitomaa J (2004). Phylogenetic evidence for the early evolution of microcystin synthesis.. Proc Natl Acad Sci U S A.

[10] Rounge TB, Rohrlack T, Tooming-Klunderud A, Kristensen T, Jakobsen KS (2007). Comparison of cyanopeptolin genes in *Planktothrix*, *Microcystis*, and *Anabaena* strains: evidence for independent evolution within each genus.. Appl Environ Microbiol.

[11] Ryoo IJ, Park HR, Choo SJ, Hwang JH, Park YM (2006). Selective cytotoxic activity of valinomycin against HT-29 Human colon carcinoma cells via down-regulation of GRP78.. Biol Pharm Bull.

[12] Cheng YQ (2006). Deciphering the biosynthetic codes for the potent anti-SARS-CoV cyclodepsipeptide valinomycin in *Streptomyces tsusimaensis* ATCC 15141.. Chembiochem.

[13] Magarvey NA, Ehling-Schulz M, Walsh CT (2006). Characterization of the cereulide NRPS alpha-hydroxy acid specifying modules: activation of alpha-keto acids and chiral reduction on the assembly line.. J Am Chem Soc.

[14] Agata N, Mori M, Ohta M, Suwan S, Ohtani I (1994). A novel dodecadepsipeptide, cereulide, isolated from *Bacillus cereus* causes vacuole formation in HEp-2 cells.. FEMS Microbiol Lett.

[15] Apetroaie C, Andersson MA, Sproer C, Tsitko I, Shaheen R (2005). Cereulide-producing strains of *Bacillus cereus* show diversity.. Arch Microbiol.

[16] Ehling-Schulz M, Fricker M, Grallert H, Rieck P, Wagner M (2006). Cereulide synthetase gene cluster from emetic *Bacillus cereus*: structure and location on a mega virulence plasmid related to *Bacillus anthracis* toxin plasmid pXO1.. BMC Microbiol.

[17] Rasko DA, Rosovitz MJ, Okstad OA, Fouts DE, Jiang L (2007). Complete sequence analysis of novel plasmids from emetic and periodontal *Bacillus cereus* isolates reveals a common evolutionary history among the *B. cereus*-group plasmids, including *Bacillus anthracis* pXO1.. J Bacteriol.

[18] Li W-H (1997). Molecular Evolution..

[19] Hillis DM, Moritz C, Mable BK, editors (1996). Molecular Systematics. 2nd ed..

[20] Darlu P, Lecointre G (2002). When does the incongruence length difference test fail?. Mol Biol Evol.

[21] Bentley SD, Chater KF, Cerdeno-Tarraga AM, Challis GL, Thomson NR (2002). Complete genome sequence of the model actinomycete *Streptomyces coelicolor* A3(2).. Nature.

[22] Gaschen B, Kuiken C, Korber B, Foley B (2001). Retrieval and on-the-fly alignment of sequence fragments from the HIV database.. Bioinformatics.

[23] Perkins JB, Guterman SK, Howitt CL, Williams VE,, Pero J (1990). *Streptomyces* genes involved in biosynthesis of the peptide antibiotic valinomycin.. J Bacteriol.

[24] Anderson AS, Wellington EM (2001). The taxonomy of *Streptomyces* and related genera.. Int J Syst Evol Microbiol.

[25] Huddleston AS, Cresswell N, Neves MC, Beringer JE, Baumberg S (1997). Molecular detection of streptomycin-producing streptomycetes in Brazilian soils.. Appl Environ Microbiol.

[26] Andersson MA, Mikkola R, Kroppenstedt RM, Rainey FA, Peltola J (1998). The mitochondrial toxin produced by *Streptomyces griseus* strains isolated from an indoor environment is valinomycin.. Appl Environ Microbiol.

[27] Martiny JB, Bohannan BJ, Brown JH, Colwell RK, Fuhrman JA (2006). Microbial biogeography: putting microorganisms on the map.. Nat Rev Microbiol.

[28] Brown EW, LeClerc JE, Kotewicz ML, Cebula TA (2001). Three R's of bacterial evolution: how replication, repair, and recombination frame the origin of species.. Environ Mol Mutagen.

[29] Wiener P, Egan S, Huddleston AS, Wellington EM (1998). Evidence for transfer of antibiotic-resistance genes in soil populations of streptomycetes.. Mol Ecol.

[30] Kieser T, Bibb MJ, Buttner MJ, Chater KF, Hopwood DA (2000). Practical *Streptomyces* Genetics..

[31] Thompson JD, Higgins DG, Gibson TJ (1994). CLUSTAL W: improving the sensitivity of progressive multiple sequence alignment through sequence weighting, position-specific gap penalties and weight matrix choice.. Nucleic Acids Res.

[32] Maddison DR, Swofford DL, Maddison WP (1997). NEXUS: an extensible file format for systematic information.. Syst Biol.

[33] Maddison DR, Maddison WP (2001). MacClade 4, v. 4.01..

[34] Cole JR, Chai B, Farris RJ, Wang Q, Kulam SA (2005). The Ribosomal Database Project (RDP-II): sequences and tools for high-throughput rRNA analysis.. Nucleic Acids Res.

[35] Swofford DL (2003). PAUP* Phylogenetic Analysis Using Parsimony (* and Other Methods)..

[36] Kitching IJ, Forey PL, Humphries CJ, Williams D (1998). Cladistics: theory and practice of parsimony analysis..

[37] Farris JS, Kallersjo M, Kluge AG, Bult C (1995). Testing significance of incongruence.. Cladistics.

[38] Huelsenbeck JP, Ronquist F (2001). MRBAYES: Bayesian inference of phylogenetic trees.. Bioinformatics.

[39] Nylander JA (2004). *MrModelTest v2. Program distributed by the author*..

[40] Felsenstein J (1981). Evolutionary trees from DNA sequences: a maximum likelihood approach.. J Mol Evol.

[41] Page RDM, Holmes EC (1998). Molecular Evolution: A Phylogenetic Approach..

[42] Zwickl DJ (2006). Genetic algorithm approaches for the phylogenetic analysis of large biological sequence datasets under the maximum likelihood criterion. Ph.D. dissertation [Ph.D. dissertation]..

[43] McInerney JO (1998). GCUA: general codon usage analysis.. Bioinformatics.

[44] Nishimura H (1968). Method of controlling rice blast..

[45] Taber WA, Vining LC (1957). Amidomycin, a new antibiotic from a streptomyces species; production, isolation, assay, and biological properties.. Can J Microbiol.

[46] Pettit GR, Tan R, Melody N, Kielty JM, Pettit RK (1999). Antineoplastic agents. Part 409: Isolation and structure of montanastatin from a terrestrial actinomycete.. Bioorg Med Chem.

[47] Brockmann H, Schmidt-Kastner G (1955). Valinomycin I. XXVII. Mittail über Antibiotica aus Actinomyceten.. Chem Ber.

[48] Michel KH, Kastner RE (1985). A54556 antibiotics and process for production thereof..

