# Supplementary material for: Valinomycin Biosynthetic Gene Cluster in Streptomyces: Conservation, Ecology and Evolution
Source: PLoS One. 2009 Sep 29;4(9):e7194. doi: 10.1371/journal.pone.0007194 (PMC2746310; doi:10.1371/journal.pone.0007194)
Supplement: Table S1 — Primers used to amplify the VLM biosynthetic gene (vlm) fragments, 16S rDNA and trpB. (0.08 MB DOC) [file pone.0007194.s001.doc]

**Table S1. Primers used to amplify the VLM biosynthetic gene (*vlm*) fragments, 16S rDNA and *trpB*.** Only the primers used in generating data reported in this paper are listed here. P = PCR amplification, S = sequencing.

| **Target Gene**  **(Amplicon)** | **Primer** | **5’ to 3’ Sequence** | **Use** | **Tm (ºC)** | **Reference or Note** | **Amplicon Size (bp)^1^** |
| --- | --- | --- | --- | --- | --- | --- |
| *vlm1*(G) | VLM1-F1  VLM1-F3  VLM1-RP2  VLM1-581F | ACCTCGGGGAGCACGGGAG  GGGAGCACGGGAGCGCCCAA  TGGCCCGCTGGATCTTGCC  AATTCGTCGCGCTSGGCCG | P, S  P, S  P, S  S | 60  62  58  58 | This study  This study  This study  This study | 1110  1104  ----  N/A |
| *vlm1/2* (H) | VLM1/2-F  VLM1/2-R  VLM1/2-567R  VLM1/2-1081F  VLM1/2-1516F  Contig12-FP3  Contig12-RP3 | GACTGCTCATCAACACGCTGC  TGCCGGTGCTCCCCGAGGT  GCTGGTCCTCGTGCTCGGC  CACCGTCTCCTGCCTGCCG  CGGGTGTCGGCCTTCCTCCG  TCGAATTCTTCGAGAAGTTCCG  GGGAGTCGTACGACTACGG | P, S  P, S  S  S  S  S  S | 56  60  60  60  62  53  55 | This study  This study  This study  This study  This study  This study  This study | 1827  ----  N/A  N/A  N/A  N/A  N/A |
| *vlm2* (I) | VLM2-F  VLM2-R  VLM2-423F | TCCTCGGCTACATCGAACTGG  CGTGGTCCCCGGGGATGTC  GACCGGGCGAAGAACGATC | P, S  P, S  S | 56  60  55 | This study  This study  This study | 668  ----  N/A |
| 16S rDNA | 16S-27F  16S-1492R  16S-515F  16S-621R  16S-785R  16S-1036F | AAGAGTTTGATCMTGGCTCA  TACGGYTACCTTGTTACGACTT  GTGCCAGCMGCCGCGGTAA  CGTATCGAKTGCAGACCCGG  GACTACCAGGGTATCTAATCC  CAGGTGGTGCATGGCTGTCG | P, S  P, S  S  S  S  S | 49  52  59  57  52  58 | [1]  [1]  This study  This study  [2]  This study | 1465  ----  N/A  N/A  N/A  N/A |
| *trpB* | trpB-F  trpB-R | CGATCTCGGCCGGTCTGGACTA  CACCGGGTCGCTGTGCGGC | P, S  P, S | 60  62 | [3]  [3] | 264  ---- |

**^1^** Refer to DNA sizes amplified from the reference strain *S. tsusimaensis*.

References:

1. Lane DJ (1991) Nucleic acid techniques in bacterial systematics; Stackebrandt E, Goodfellow M, editors. New York: Wiley & Sons. 115-175 p.

2. Amann RI, Ludwig W, Schleifer KH (1995) Phylogenetic identification and in situ detection of individual microbial cells without cultivation. Microbiol Rev 59: 143-169.

3. Huddleston AS, Cresswell N, Neves MC, Beringer JE, Baumberg S, et al. (1997) Molecular detection of streptomycin-producing streptomycetes in Brazilian soils. Appl Environ Microbiol 63: 1288-1297.
