# Supplementary material for: Valinomycin Biosynthetic Gene Cluster in Streptomyces: Conservation, Ecology and Evolution
Source: PLoS One. 2009 Sep 29;4(9):e7194. doi: 10.1371/journal.pone.0007194 (PMC2746310; doi:10.1371/journal.pone.0007194)
Supplement: Table S2 — Strains and GenBank accession numbers of DNA sequences used for phylogenetic analyses. (0.14 MB DOC) [file pone.0007194.s002.doc]

**Table S2. Strains and GenBank accession numbers of DNA sequences used for phylogenetic analyses.** GenBank accession numbers by bold-face type indicate sequences acquired by this study or our previous study [1]; others were obtained from the Ribosomal Database Project (RDP-II) [2] or from the GenBank database.

| **Strain** | **16S rDNA** | ***vlm1*** | ***vlm1/2*** | ***vlm2*** | ***trpB*** |
| --- | --- | --- | --- | --- | --- |
| *Bacillus cereus* AH187 | CP001177 | DQ889676  (*cesA*) | DQ889676  (*cesA/B*) | DQ889676  (*cesB*) | CP001177 |
| *Mycobacterium tuberculosis* H37Rv | X58890 |  |  |  | CAB08906 |
| *Nocardia farcinica* IFM 10152 | AP006618 |  |  |  | BAD56707 |
| *Streptomyces albidoflavus* 1886 | AJ002090 |  |  |  |  |
| *Streptomyces albidoflavus* DSM 40792 | Z76677 |  |  |  |  |
| *Streptomyces albus* JCM 10204 | AB045884 |  |  |  |  |
| *Streptomyces anulatus* (Malaysia) | **EU647478** | **EU647479** | **EU647480** | **EU647481** | **EU647482** |
| *Streptomyces anulatus* (Montana) | **EU647474** | **EU647475** | **EU647476** | **EU647477** | **EU624143** |
| *Streptomyces antibioticus* NRRL B-1701 | AY999776 |  |  |  |  |
| *Streptomyces atroolivaceus* LMG 19306 | AJ781320 |  |  |  |  |
| *Streptomyces aureofaciens* IMET 43577 | AY289116 |  |  |  |  |
| *Streptomyces bikiniensis* DSM 40581 | X79851 |  |  |  |  |
| *Streptomyces blastmyceticus* NRRL B-5480 | AY999802 |  |  |  |  |
| *Streptomyces chromofuscus* NRRL B-12175 | AY999800 |  |  |  |  |
| *Streptomyces chromogenus* NBRC 13374 | AB184362 |  |  |  |  |
| *Streptomyces clavuligerus* JCM 4710 | AB045869 |  |  |  |  |
| *Streptomyces coelicolor* A3(2) M145 | AL939116 |  |  |  | CAB51429 |
| *Streptomyces cyaneus* ISP 5106 | AJ399470 |  |  |  |  |
| *Streptomyces diastaticus* NBRC 13412 | AB184386 |  |  |  |  |
| *Streptomyces exfoliatus* (Malaysia) | **EU647483** | **EU647484** | **EU647485** | **EU647486** | **EU647487** |
| *Streptomyces exfoliatus* NRRL B-1237 | AY999796 |  |  |  |  |
| *Streptomyces fradiae* NBRC 12174 | AB184059 |  |  |  |  |
| *Streptomyces fulvissimus* DSM 40767 | **EU647488** | **EU647489** | **EU647490** | **EU647491** | **EU647492** |
| *Streptomyces fulvissimus* NBRC 13482 | AB184434 |  |  |  |  |
| *Streptomyces glaucescens* DSM 40716 | X79322 |  |  |  |  |
| *Streptomyces griseoflavus* LMG 19344 | AJ781322 |  |  |  |  |
| *Streptomyces griseoruber* NRRL B-1818 | AY999723 |  |  |  |  |
| *Streptomyces griseoviridis* NBRC 12762 | AB184125 |  |  |  |  |
| *Streptomyces griseus* 1/k DSM 41748 | **EU647493** | **EU647494** | **EU647495** | **EU647496** | **EU647497** |
| *Streptomyces griseus* 10/ppi DSM 41751 | **EU647498** | **EU647499** | **EU647500** | **EU647501** | **EU647502** |
| *Streptomyces hawaiiensis* NRRL 15010 | **EU624140** |  |  |  | **EU624141** |
| *Streptomyces lavendulae* NBRC 12340 | AB184077 |  |  |  |  |
| *Streptomyces longisporoflavus* NBRC 12886 | AB184220 |  |  |  |  |
| *Streptomyces lydicus* ATCC 25470 | Y15507 |  |  |  |  |
| *Streptomyces microflavus* NBRC 13062 | AB184284 |  |  |  |  |
| *Streptomyces netropsis* LMG 20320 | AJ781375 |  |  |  |  |
| *Streptomyces nogalater* NBRC 13445 | AB184408 |  |  |  |  |
| *Streptomyces olivaceovirdis* NBRC 13066 | AB184288 |  |  |  |  |
| *Streptomyces phaeochromogens* DSM 40073 | AF500071 |  |  |  |  |
| *Streptomyces poonensis* NBRC 12556 | AB184103 |  |  |  |  |
| *Streptomyces purpureus* NBRC 13927 | AB184547 |  |  |  |  |
| *Streptomyces rimosus* JCM 4667 | AB045883 |  |  |  |  |
| *Streptomyces roseoverticillatus* LMG 20433 | AJ781381 |  |  |  |  |
| *Streptomyces roseus* NBRC 12818 | AB184879 |  |  |  |  |
| *Streptomyces* sp. PRL 1642 ATCC 23836 | **EU647470** | **EU647471** | **EU647472** | **EU647473** | **EU624142** |
| *Streptomyces thermovulgaris* DSM 40579 | Z68098 |  |  |  |  |
| *Streptomyces tsusimaensis* ATCC 15141 | **EU622279** | **DQ174261** | **DQ174261** | **DQ174261** | **EU622280** |
| *Streptomyces violaceus* ISP 5209 | AJ399480 |  |  |  |  |
| *Streptomyces violaceusniger* NRRL B-1476T | AJ391822 |  |  |  |  |
| *Streptomyces viridochromogens* JCM 5013 | AB045858 |  |  |  |  |

References:

1. Cheng YQ (2006) Deciphering the biosynthetic codes for the potent anti-SARS-CoV cyclodepsipeptide valinomycin in *Streptomyces tsusimaensis* ATCC 15141. Chembiochem 7: 471-477.

2. Cole JR, Chai B, Farris RJ, Wang Q, Kulam SA, et al. (2005) The Ribosomal Database Project (RDP-II): sequences and tools for high-throughput rRNA analysis. Nucleic Acids Res 33: D294-296.
