# Supplementary material for: Valinomycin Biosynthetic Gene Cluster in Streptomyces: Conservation, Ecology and Evolution
Source: PLoS One. 2009 Sep 29;4(9):e7194. doi: 10.1371/journal.pone.0007194 (PMC2746310; doi:10.1371/journal.pone.0007194)
Supplement: Table S3 — Primary data of phylogenetic analyses. (0.09 MB DOC) [file pone.0007194.s003.doc]

**Table S3. Primary data of phylogenetic analyses. CI = consistency index, PHT = partition homogeneity test, RC = rescaled consistency index, RI = retention index. N = nucleotide (DNA) sequence, P = protein (amino acid) sequence.**

| **Data sets** | ***vlm1*** | ***vlm1/2*** | ***vlm2*** | ***vlm*-** **Combined** | **Vlm1** | **Vlm1/2** |
| --- | --- | --- | --- | --- | --- | --- |
| *Data*  Type  Alignment length  Variable characters  Indels | N  1045  180  2 | N  1847  356  1 | N  658  87  0 | N  3550  623  3 | P  347  68  2 | P  606  130  1 |
| *MP analysis*  Informative characters  Number of trees  Tree length  CI  RI  RC  PHT | 155  1  219  0.937  0.954  0.894  N/A | 263  1  428  0.906  0.924  0.838  N/A | 79  1  110  0.922  0.940  0.866  N/A | 497  1  758  0.917  0.935  0.857  0.250 | 59  1  85  0.947  0.961  0.910  N/A | 98  1  164  0.946  0.956  0.904  1.000 |
| **Data sets** | **Vlm2** | **Vlm- Combined** | ***Strep* 16S**  **(49 taxa)** | **16S**  **(11 taxa)** | ***trpB*** | **16S/*trpB*-Combined** |
| *Data*  Type  Alignment length  Variable characters  Indels | P  219  22  0 | P  1172  220  3 | N  1413  284  5 | N  1428  167  4 | N  264  74  0 | N  1692  241  4 |
| *MP analysis*  Informative characters  Number of trees  Tree length  CI  RI  RC  PHT | 20  1  27  0.960  0.971  0.933  N/A | 177  2  277  0.943  0.956  0.902  0.943 | 203  99  866  0.365  0.695  0.253  N/A | 39  1  208  0.746  0.709  0.529  N/A | 30  7  93  0.660  0.698  0.460  N/A | 69  5  307  0.677  0.636  0.430  0.010 |
| **Data set** | **TrpB** | **All-Combined** |  |  |  |  |
| *Data*  Type  Alignment length  Variable characters  Indels | P  106  36  0 | N  5242  138  6 |  |  |  |  |
| *MP analysis*  Informative characters  Number of trees  Tree length  CI  RI  RC  PHT | 11  7  43  0.800  0.824  0.659  N/A | 518  1  794  0.915  0.934  0.854  0.405 |  |  |  |  |
