# Supplementary material for: Valinomycin Biosynthetic Gene Cluster in Streptomyces: Conservation, Ecology and Evolution
Source: PLoS One. 2009 Sep 29;4(9):e7194. doi: 10.1371/journal.pone.0007194 (PMC2746310; doi:10.1371/journal.pone.0007194)
Supplement: Table S4 — Genetic distances calculated between concatenated vlm (Streptomyces) and ces (Bacillus cereus) DNA sequences. (0.05 MB DOC) [file pone.0007194.s004.doc]

**Table S4. Genetic distances calculated between concatenated *vlm* (*Streptomyces*) and *ces* (*Bacillus cereus*) DNA sequences.** The F81 algorithm [1] was used for calculation. Strain names are represented by taxon numbers: 0, *Bacillus cereus* AH187; 1; S. TSUSI (JAP); 2, S. spPRL (CAN); 3, S. ANULA (USA); 4, S. ANULA (MAL); 5, S. EXFOL (MAL); 6, S. FULVI (GER); 7, S. GRIS1 (FIN); S. GRIS2 (FIN) (by acronyms; see table 1 for details).

**Query Sequence Target Sequence Genetic Distance**

vlm taxon7 vlm taxon6 0.00815629

vlm taxon3 vlm taxon6 0.01138836

vlm taxon3 vlm taxon7 0.01079574

vlm taxon2 vlm taxon6 0.09731667

vlm taxon2 vlm taxon7 0.09864549

vlm taxon2 vlm taxon3 0.09400701

vlm taxon8 vlm taxon6 0.09626751

vlm taxon8 vlm taxon7 0.09759436

vlm taxon8 vlm taxon3 0.09295649

vlm taxon8 vlm taxon2 0.00894905

vlm taxon5 vlm taxon6 0.09592208

vlm taxon5 vlm taxon7 0.09659276

vlm taxon5 vlm taxon3 0.09229041

vlm taxon5 vlm taxon2 0.04003507

vlm taxon5 vlm taxon8 0.03614748

vlm taxon1 vlm taxon6 0.12701593

vlm taxon1 vlm taxon7 0.12629430

vlm taxon1 vlm taxon3 0.12425570

vlm taxon1 vlm taxon2 0.12306301

vlm taxon1 vlm taxon8 0.12163305

vlm taxon1 vlm taxon5 0.11916509

vlm taxon4 vlm taxon6 0.12776969

vlm taxon4 vlm taxon7 0.12773719

vlm taxon4 vlm taxon3 0.12604223

vlm taxon4 vlm taxon2 0.12451079

vlm taxon4 vlm taxon8 0.12375588

vlm taxon4 vlm taxon5 0.12164185

vlm taxon4 vlm taxon1 0.02170665

ces taxon0 vlm taxon6 0.98676306

ces taxon0 vlm taxon7 0.98971123

ces taxon0 vlm taxon3 0.98670727

ces taxon0 vlm taxon2 0.97571105

ces taxon0 vlm taxon8 0.98111689

ces taxon0 vlm taxon5 0.97379059

ces taxon0 vlm taxon1 0.95608395

ces taxon0 vlm taxon4 0.96656400

Reference:

1. Felsenstein J (1981) Evolutionary trees from DNA sequences: a maximum likelihood approach. J Mol Evol 17: 368-376.
