# Supplementary material for: Valinomycin Biosynthetic Gene Cluster in Streptomyces: Conservation, Ecology and Evolution
Source: PLoS One. 2009 Sep 29;4(9):e7194. doi: 10.1371/journal.pone.0007194 (PMC2746310; doi:10.1371/journal.pone.0007194)
Supplement: Table S5 — G+C content and substitution rates for the vlm data sets. (0.04 MB DOC) [file pone.0007194.s005.doc]

**Table S5. G + C content and substitution rates for the *vlm* data sets.** ds = Jukes-Cantor correction for observed synonymous substitutions, dn: Jukes-Cantor correction for observed non-synonymous substitutions.

| **Data Set** | **(G + C)%** | **ds** | **dn** | **ds/dn** |
| --- | --- | --- | --- | --- |
| *vlm1* | 69.0 | 0.06 | 0.11 | 0.60 (0.25) |
| *vlm1/2* | 71.0 | 0.14 | 0.08 | 1.72 (0.23) |
| *vlm2* | 67.0 | 0.09 | 0.07 | 1.34 (0.20) |
| *vlm*-Comb | 70.0 | 0.12 | 0.08 | 1.57 (0.35) |
