# Supplementary figures and images for: Valinomycin Biosynthetic Gene Cluster in Streptomyces: Conservation, Ecology and Evolution
Source: PLoS One. 2009 Sep 29;4(9):e7194. doi: 10.1371/journal.pone.0007194 (PMC2746310; doi:10.1371/journal.pone.0007194)

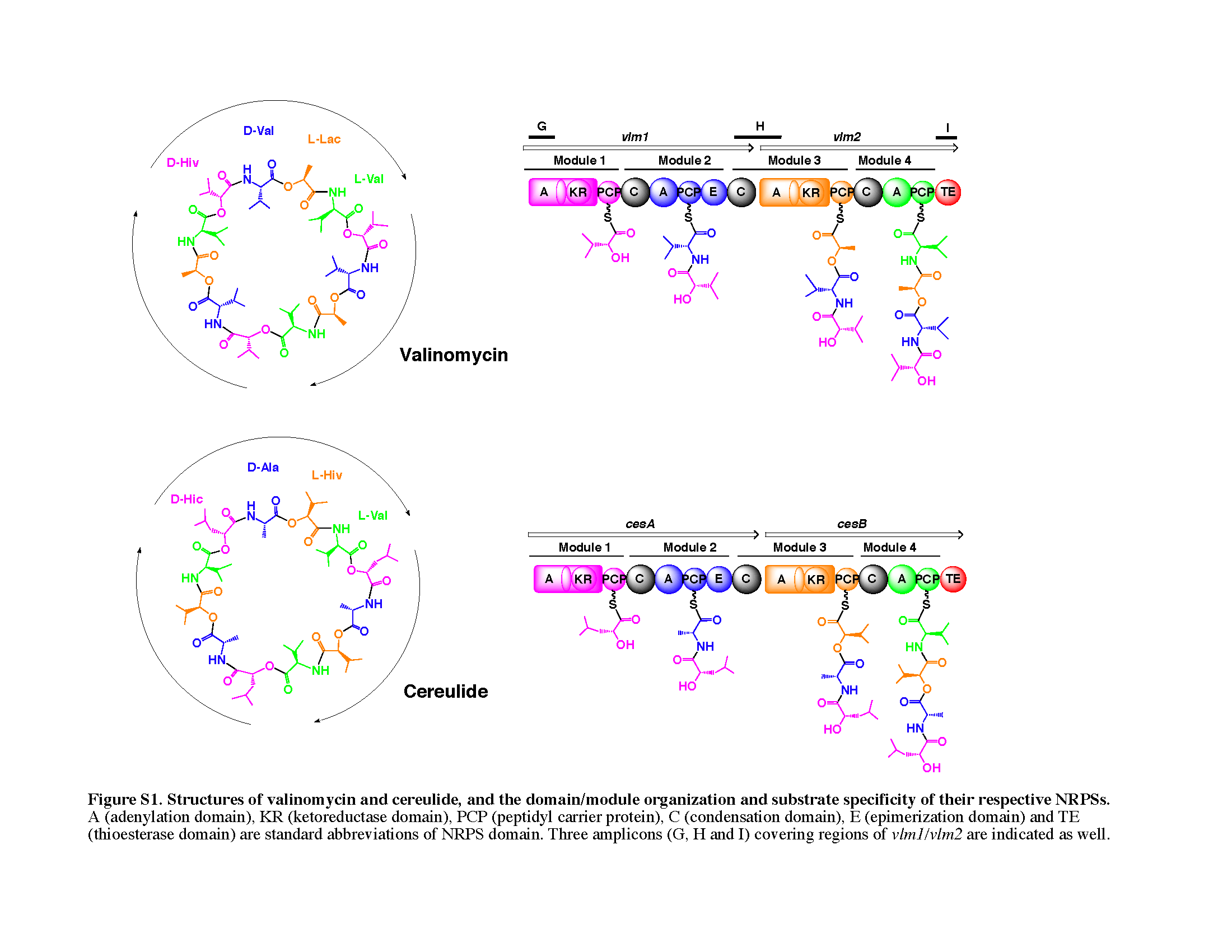

Supplement: Figure S1 — Structures of valinomycin and cereulide, and the domain/module organization and substrate specificity of their respective NRPSs. (0.40 MB TIF) [file pone.0007194.s006.tif]

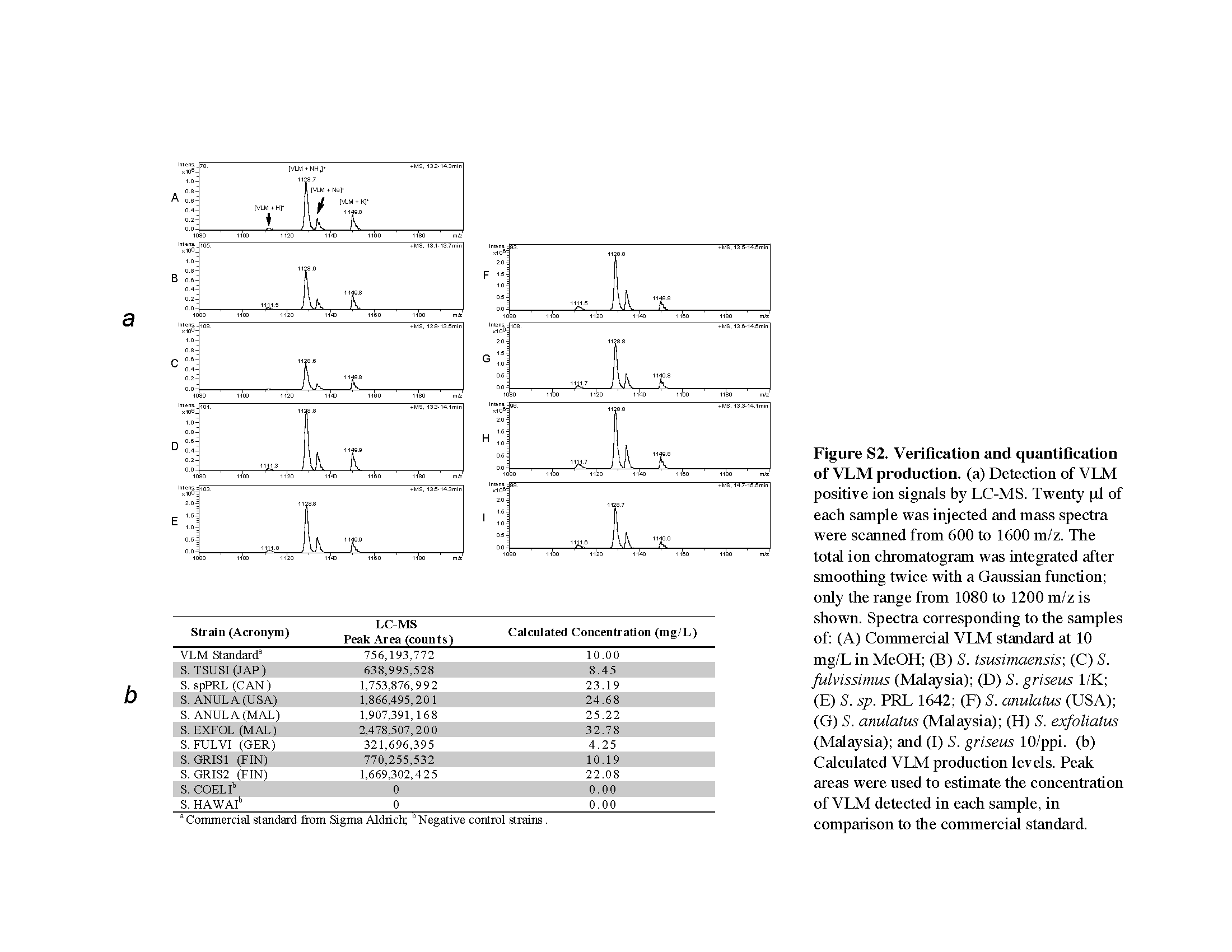

Supplement: Figure S2 — Verification and quantification of VLM production. (0.13 MB TIF) [file pone.0007194.s007.tif]

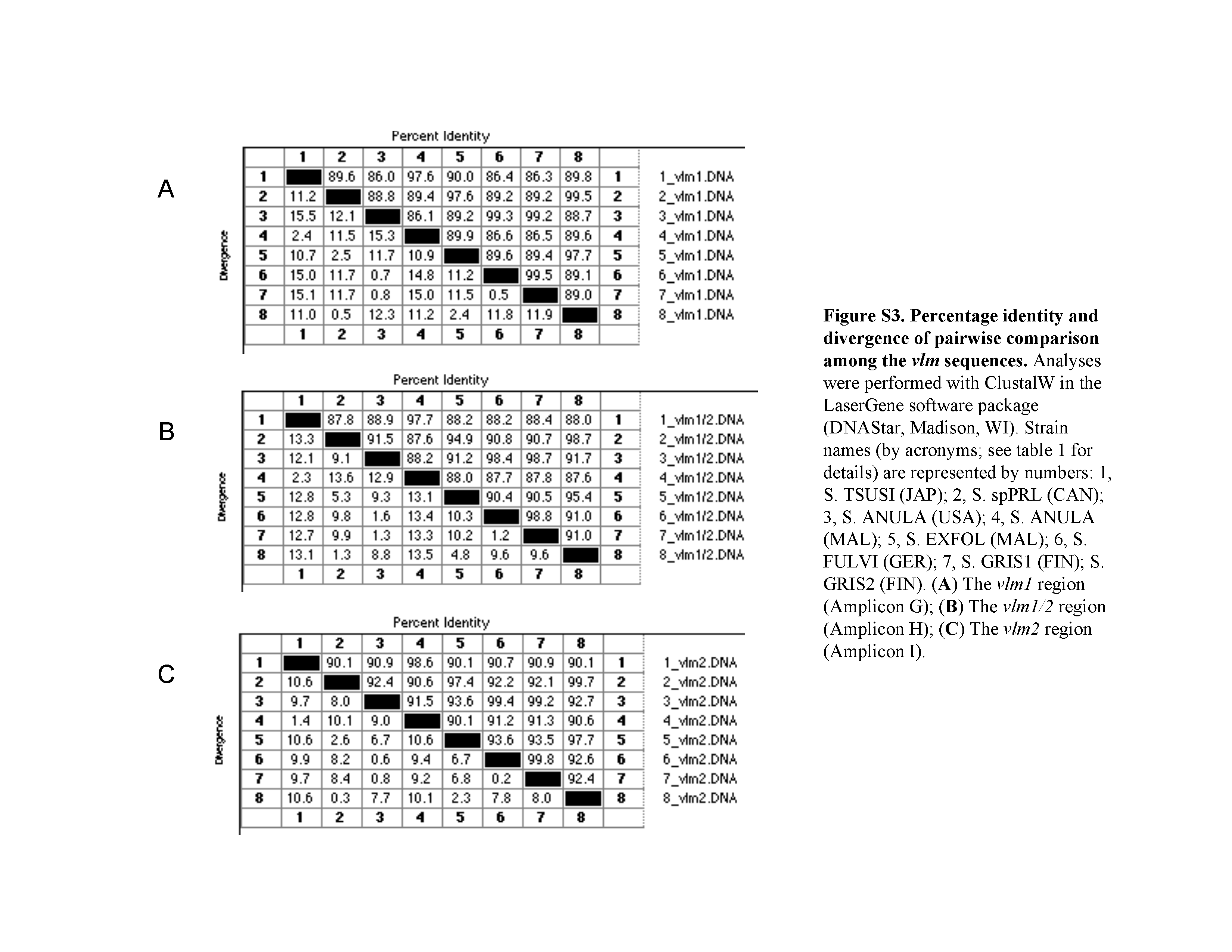

Supplement: Figure S3 — Percentage identity and divergence of pairwise comparison among the vlm sequences. (1.32 MB TIF) [file pone.0007194.s008.tif]
